# The Limitations of Collagen/CPP Hybrid Peptides as Carriers for Cancer Drugs to FaDu Cells

**DOI:** 10.3390/molecules24040676

**Published:** 2019-02-14

**Authors:** Kevin Ho, Cristobal Morfin, Katarzyna Slowinska

**Affiliations:** Department of Chemistry and Biochemistry, California State University, Long Beach, 1250 Bellflower Blvd, Long Beach, CA 90840, USA; kevinqho@gmail.com (K.H.); cmorfim@gmail.com (C.M.)

**Keywords:** collagen peptides, cell penetrating peptides, hybrid peptides, FaDu, drug delivery, cell surface, HSPG

## Abstract

The in vitro efficacy of cancer prodrugs varies significantly between malignant cell lines. The most commonly identified problems relate to delivery: uptake mechanism, endosomal entrapment, and drug release. Here we present the study of collagen/cell penetrating hybrid (COL/CPP) peptide carriers intended to deliver paclitaxel to the hypopharyngeal carcinoma (FaDu) cells. Confocal microscopy imaging revealed the surprising response of FaDu cell to COL/CPP in comparison to previously studied cancer cell lines: hybrid peptides that carry both COL and CPP domain adsorb on the FaDu cell surface. While the CPP domain was design to facilitate the cellular uptake, in the case of FaDu cells, it also induced detrimental interactions with the cell membrane. Despite surface adsorption, the colocalization study with endosomal markers EEA1 and LAMP1 reveals that COL/CPP is internalized via endosomal pathway, peptides are able to escape before lysosome formation and release paclitaxel. Therefore, the main obstacle for paclitaxel delivery to FaDu cells appears to be related to cell surface properties. This behavior seems specific to FaDu cells, and could be linked to previously reported overexpression of T5, heparanase splice variants that produces protein lacking enzymatic activity of heparanase. This results in increased concentration of HSPG on FaDu cell surface, and possibly creates a barrier for cellular uptake of highly charged COL/CPP.

## 1. Introduction

Despite numerous oncological advancements, cancer is the second leading cause of death in the United States [1]. Chemotherapy together with surgery, radiation, immuno-, and hormonal therapy are among the available treatment options today [2]. Frequently, the localization of the cancer determines the course of therapy. In head and neck cancers (HNCs) that affect the pharynx—an organ essential to the ability to swallow and to speak—surgery is often problematic, particularity in the late stages of the cancer [3,4]. Due to the significant patient morbidity associated with surgical treatment and an increasing trend towards organ preservation strategies, treatments with an emphasis on organ preservation and restoration have become more common [5,6]. Thus, chemotherapy is preferential. A treatment regime using paclitaxel as one of the drugs was shown to have superior survival rates in HNC patients, compared to those only treated with cisplatin and fluorouracil [6].

Paclitaxel (PTX) is a very potent chemotherapy drug, but its hydrophobic character causes a major problem with administration due to its low aqueous solubility [7]. To increase the solubility and improve the paclitaxel administration, the chemical modifications or changes in formulations were proposed in numerus reports [8,9,10]. Previously, we reported the conjugation of paclitaxel to a hybrid collagen-cell penetrating peptide (COL/CPP) carrier and its biological characterization [11].

The COL/CPP peptide is a hybrid consisting of two functional domains: cell penetrating peptide and collagen domain. Cell penetrating peptides (CPP) are short peptides that can penetrate cellular membranes and assist in the cellular internalization of biologically relevant cargo [12]. They were first discovered as a short sequence of TAT protein [13]. Although cationic CPPs are one of the best molecular transporters, their use has limitations [14]. Their large positive charge can lead to diminished cell viability and disrupted membrane integrity upon cellular uptake [15,16,17]. In addition, the susceptibility of peptides to enzymatic degradation limits the circulation time upon administration. Previously, we have shown that modifying the CPP peptide with the structural collagen domain (COL) solves many of the problems associated with CPPs. The COL domain consists of several repeats of (Pro-Hyp-Gly)_n_ sequence, where *n* determines the transition temperature of the collagen domain folding into a triple helix. The introduction of the collagen folding domain allows the peptide to reversibly fold into rigid nanoparticle, which improves resistance to enzymatic degradation [18].

We have shown in the past investigations that COL/CPP peptide conjugated to PTX forms an effective drug delivery system for acute T-cell leukemia (Jurkat cells), IC_50_ = 27 nM, but decreases in effectiveness for lung carcinoma (A549 cells), IC_50_ = 7.5 µM [11]. The difference in efficacy was attributed to the endosomal entrapment that was present in A549, but not in Jurkat cells.

The hypopharyngeal squamous cell carcinoma cell line FaDu represents a good model of the HNCs [5]. Here we examined the possibility of COL/CPP application as a potential carrier to deliver cancer drugs to FaDu cells. While we observed an acceptable IC_50_ of paclitaxel delivered to FaDu cells (0.58 μM) with COL/CPP carrier, it is far from low-nanomolar range expected for paclitaxel [7]. Confocal microscopy was employed to determine the cause of lower efficacy of the paclitaxel which is most likely related to delivery problems. We have shown that the COL/CPP peptide is uptaken by endosomal pathway, but manages to escape before the conversion of endosome to lysozyme. Thus, the problems with delivery to lung carcinoma cells (A549) observed in the past are not present in FaDu cells [11]. Closer examination of the FaDu cells showed an unusual interaction of the peptides with the cell surface membrane. We proposed that this interaction is related to the increased concentration of heparan sulfate proteoglycans (HSPG) on the cell surface that is not present in other cell lines we studied in the past [19]. HSPGs often function as docking sites for protein and peptides, and it is likely that HSPG would promote COL/CPP adhesion to the cell surface [19,20]. This hypothesis is also supported by previously reported overexpression of T5, heparanase splice variants in FaDu cells, which produces protein lacking enzymatic activity of heparanase, and thus prevents cleavage of HS form HSPG [21,22].

## 2. Results

### 2.1. Hybrid Peptides

Peptides in this study were synthesized, purified, and characterized (HPLC and MS) by the Tufts University Core Facility, with exception of PTX8V1, where conjugation of the peptide to paclitaxel was performed in house. The details of the bioconjugation reaction and characterization is described elsewhere [11]. The sequences of all studied peptides are listed in Table 1 and the domains (collagen and cell penetrating) are indicated. All peptides were modified with the fluorescence tag fluorescein (FL) at the N-terminus via BaGG (Ba represents β-alanine) linker to prevent fluorescence quenching. The C-terminus was protected by amidation to prevent unwanted interactions. The *N*-terminus of PTX8V1 was protected by acylation, and in the middle of the collagen domain a PKG sequence was inserted, so the primary amine located at K14 can be used as a sole modification site for conjugation of PTX modified with an esterified succinate linker at the C2′-OH position.

All studied peptides have a collagen domain that determines the conformation of the peptide [23,24]. The shorter collagen domain observed in FL6V1 prevents the peptide from folding into the triple helical conformation at 37 °C; thus at physiological conditions those peptides assume a random coil conformation [18]. In addition, the peptide FLV2R cannot assume the triple helical conformation due to the scrambled sequence of its collagen domain. To assist in peptide transport across the cell membrane, two CPP domains were examined: RRGRRG and R_6_. The former carries a smaller charge that assists in translocation and with every third residue in its sequence being a glycine, promotes helix propensity [24].

In the past studies, we established that only peptides that have a CPP domain and are folded into helical nanoparticles are able to cross cell membranes [18]. This conclusion was supported by studies performed on multiple cell lines; the underlying principle of this effect was based on the electrostatic argument that is well established in the CPP studies: at least nine arginines are needed to allow the peptide to cross the cell membrane [25,26]. In the case of hybrid peptides, the equivalence of +9 charge can be only realized upon peptide folding, in the triple helical conformation peptide carrying (RRG)_3_ CPP domain will accumulate +12 charge, while peptides that carry the R_6_ CPP domain will accumulate +18 charge after folding [18].

In addition to their collagen and cell penetrating domains, the peptides carry a reporter domain: either fluorescein for microscopy observations, or paclitaxel as a model drug for efficacy comparisons.

### 2.2. Hybrid Peptide as A Carrier of Model Drugs to FaDu Cells

**Figure 1:**
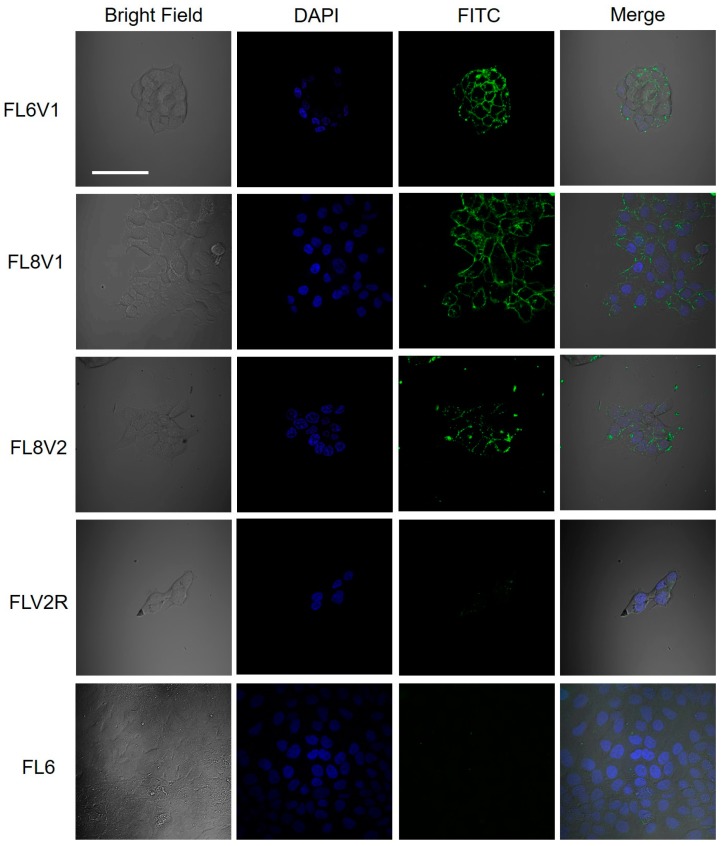
Confocal microscopy images of FaDu cells incubated for 30 min with hybrid peptides (50 μM) at 37 °C; bar represents 50 μm.

The hypopharyngeal squamous cell carcinoma (FaDu) cells were incubated with hybrid peptides (10–50 µM, 30–60 min). After the wash, living cells were stained with Hoechst stain to mark the nucleus, washed, placed in media, and imaged with confocal microscopy (Figure 1). The observed green fluorescence originates from the FITC tag present in the reporter domain of the hybrid peptides. The blue fluorescence indicates nucleus (Hoechst). The green fluorescence is observed in FaDu cells incubated with FL6V1, FL8V1, and FL8V2, but not in FaDu cells incubated with FLV2R or FL6.

### 2.3. Colocalization of Hybrid Peptide Carrier and JAM-A Protein

The initial hypothesis of hybrid peptide localization on the cell surface involved specific interactions between the hybrid peptide and cell tight junctions (TJ). The hypopharyngeal squamous cell carcinoma is characterized as being difficult to subculture during the early passages but becomes more susceptible to trypsin in later passages. The cell line is also prone to clumping and forming spheroids [27,28]. This suggests that FaDu must have a significant amount of cell–cell interactions, and thus has an upregulated expression of proteins associated with cellular TJ [29,30,31]. TJ contain several proteins: claudins, occludins, and junctional adhesion molecules (JAMs) [29,31]. The overexpression of JAM-A has been reported in several types of epithelium-derived cancers, including breast, lung, testis, and head and neck [32]. The overexpression of JAM-A also has been reported to correlate with increased incidences of metastasis in head and neck cancer [32].

**Figure 2:**
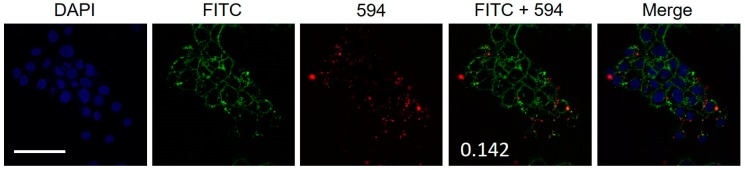
Confocal microscopy images of FaDu cells incubated for 20 min with FL8V1 (10 μM) at 37 °C and Jam-A/Alexa Fluor 594; bar represents 50 μm; number indicates Pearson’s correlation coefficient.

The FaDu cells treated with FL8V1 were subjected to immunoassay to detect colocalization of JAM-A protein in TJ and hybrid peptides. The FL8V1-treated (10 µM, 20 min) FaDu cells were incubated with rabbit anti-JAM-A primary antibody and goat anti-rabbit secondary antibody. In Figure 2, the red fluorescence originating from JAM-A and green fluorescence originating from FL8V1 are superimposed. It is well visible that they do not colocalize, and calculation of the Pearson’s correlation coefficient between the green and red fluorescence is 0.142. Thus, the null hypothesis that JAM-A and FL8V1 colocalize had to be rejected.

### 2.4. Interaction of Hybrid Peptide Carrier with FaDu Cell Surface

After determining that the hybrid peptides do not aggregate at TJ, we investigated if the hybrid peptides were interacting with the membrane of the FaDu cells through non-specific binding [33]. To test the hypothesis that the binding of hybrid peptides to FaDu cell surface is in most part driven by electrostatic interaction, we performed two experiments after cells were incubated with peptides: (1) KCL wash with high ionic strength solution and (2) competitive binding between negatively charged cell surface and negatively charged suramin present in the solution.

**Figure 3:**
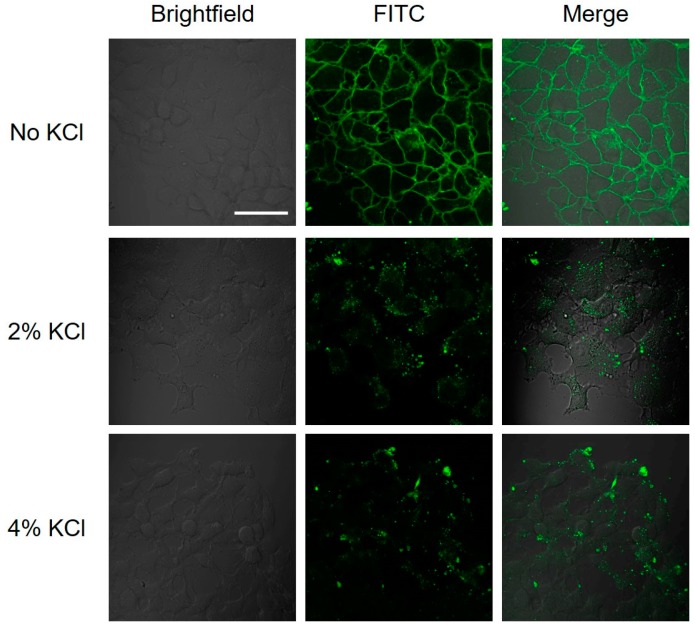
Confocal microscopy images of FL8V1-treated FaDu cells following a KCl wash. FaDu cells were incubated with 10 µM of FL8V1 at 37 °C (30 min) and washed with PBS (No KCl), 2%, and 4% KCl solution; bar represents 50 μm.

We incubated the FaDu cells with low concentration of FL8V1 (10 µM) for 20 min and treated the cells with KCl wash. The confocal images of cells treated with FL8V1 before and after a salt wash clearly indicate the peptide (green fluorescence) present on the cell surface before the wash was not present after the wash, which strongly suggests that electrostatic forces were an important component of the interactions (Figure 3). We hypothesized that it is likely that the interactions are between the hybrid peptide that carry a large positive charge and heparan sulfate proteoglycans (HSPG) expressed on the FaDu cell surface that carry negative charge. To test this hypothesis, we used the commercial heparin agarose type 1 column and tested whenever FL8V1 is retained by the column. The experiment showed that the FL8V1 peptide is retained in the heparin column and elution with 4% KCl (in 0.01 M Tris-HCl, pH = 7.5 buffer) is able to release the peptide from the column.

**Figure 4:**
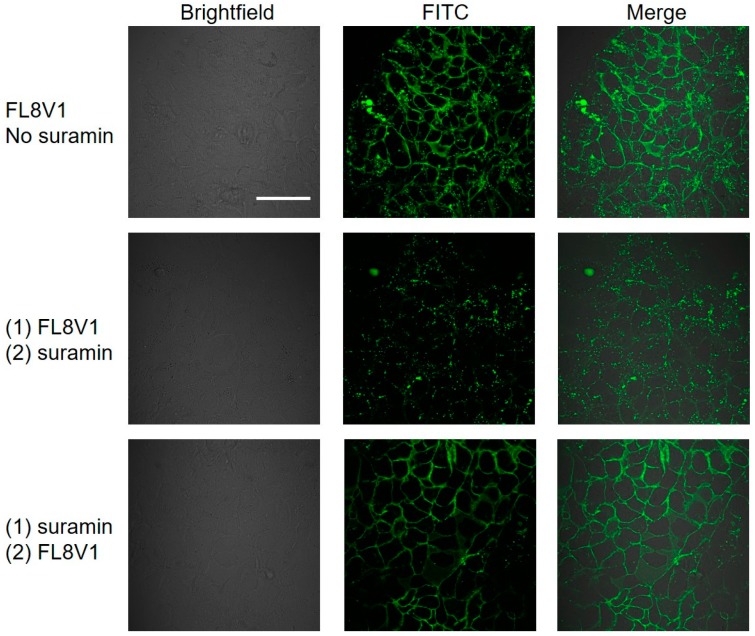
Confocal microscopy images of FL8V1-treated FaDu cells incubated for 30 min with FL8V1 (50 μM) at 37 °C without suramin treatment, post-incubation with suramin, or pre-incubation with suramin; bar represents 50 μm.

To confirm the likelihood that the cell surface interactions originate from the hybrid peptide and HS groups, we performed the experiment with suramin as a solution-based competitor that contains six sulfonic acid groups and should be able to bind hybrid peptides [34]. FaDu cells were incubated with FL8V1 peptide (50 µM, 30 min, 37 °C) and subsequently incubated with suramin (100 µM, 30 min, 37 °C). We observed the decrease of the fluorescence originating from the FL8V1 localized on the cell surface (Figure 4). When the sequence of incubation was reversed, we did not observe this effect, which demonstrates that suramin does not bind to the FaDu cell surface, but binds to FL8V1 which removes the peptide from the cell surface.

We noted that after the removal of the hybrid peptide from the cell surface, the peptides are still present in the endosome-like structures. Thus, the uptake of the peptides is not completely halted at the cell surface, and some peptides managed to be internalized.

### 2.5. Colocalization of Hybrid Peptide Carrier and Endosomal Markers

**Figure 5:**
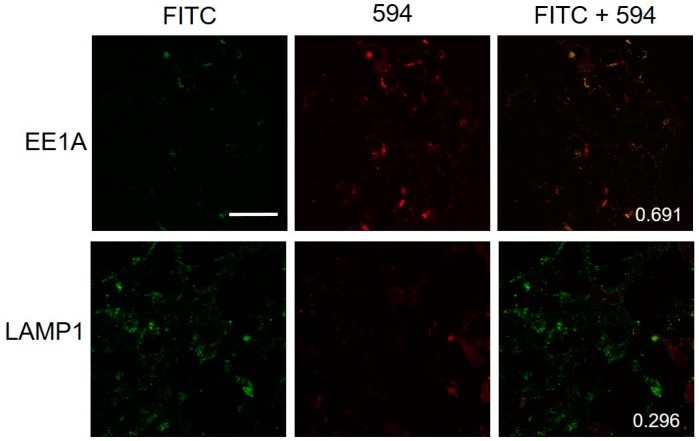
Confocal microscopy images of FaDu cells incubated for 20 min with FL8V1 (10 μM) at 37 °C and EE1A/Alexa Fluor 594 or LAMP1/Alexa Fluor 594; bar represents 50 μm; number indicates Pearson’s correlation coefficient.

To ensure that the observed structures are endosomes, two colocalization experiments were conducted to detect early endosomes and lysosomes. First, FL8V1-treated FaDu cells were incubated with EEA1 (early endosome antigen 1) antibodies. EEA1 is a protein found on early endosomes that transport endocytosed materials to late endosomes and lysosomes [35,36]. An anti-rabbit IgG secondary antibody modified with Alexa Fluor 594 was employed to image the early endosomes (red). Figure 5 shows that the spatial correlation between green fluorescence (FL8V1) and red fluorescence (EEA1/Alexa 549) is high, suggesting that the localization of hybrid peptides and localization of early endosomes overlap in FaDu cells. The Parson’s colocalization coefficient was calculated to be 0.691, which indicates the entry of hybrid peptide through an endosomal pathway.

Next, the FL8V1-treated FaDu cells were incubated with rabbit LAMP1 (lysosomal associated membrane protein 1) monoclonal antibody to detect proteins specific to late endosomes [35,36]. An anti-rabbit IgG secondary antibody modified with Alexa Fluor 594 was employed to image the late endosomes (red). In Figure 5, the green fluorescence originating from FL8V1 and the red fluorescence originating from EEA1/Alexa 549 do not show colocalization and the Pearson’s correlation coefficient was calculated to be 0.296, indicating that the peptide managed to escape from the endosome before its conversion to lysosome.

### 2.6. Efficacy of PTX8V1 in FaDu Cells

**Figure 6:**
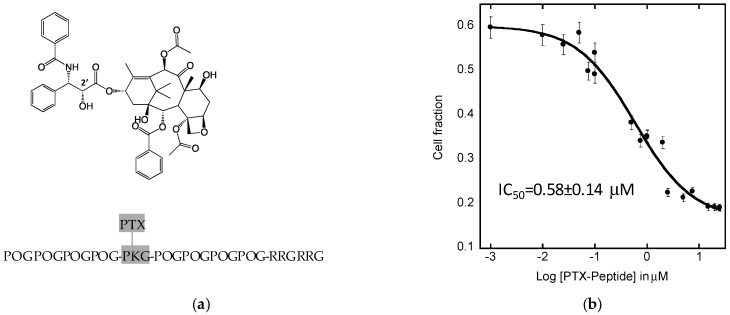
Paclitaxel (PTX) with the indicated C2′ position (modification site) and PTX8V1 with the indicated K14 conjugation site. Effect of PTX8V1 on FaDu cell survival (**a**). Error bars represent standard deviation calculated from at least 6 measurements. *R^2^* coefficient of the best fit is 0.975 (**b**).

To confirm that the peptide is able to escape from the endosome and release the drug, PTX8V1 peptide was synthesized. The detailed description of the synthesis (paclitaxel conjugation to a hybrid peptide via succinic linker) and chemical characterization of PTX8V1 is described elsewhere [11]. The FaDu cells were treated with PTX8V1 and cell viability was measured with MTT assay. The cytotoxic effects of PTX8V1 were analyzed and its IC_50_ was calculated by fitting the dose-response (sigmoidal) curves to collected data (Figure 6) using KaleidaGraph software. We have shown in the past that hybrid peptides are not cytotoxic, thus the viability of FaDu reflect the effects of paclitaxel [11]. Paclitaxel mode of action requires its presence in the cell body and the only source of PTX is PTX8V1. The measured IC_50_ was 0.58 ± 0.14 μM, which proves that PTX8V1 is internalized by FaDu cells, thus the adsorption on the cell surface does not prevent the hybrid peptide from delivering paclitaxel into the cells. In addition, low IC_50_ indicates that paclitaxel was successfully released form the prodrug.

## 3. Discussion

Collagen/CPP hybrid peptides were studied as a carrier for small molecule cancer drugs to the hypopharyngeal squamous cell carcinoma cell line FaDu. Unlike other tested cancer cells, FaDu treated with hybrid peptides showed the unique deposition of the peptides on its cell surface. We examined the hybrid peptides with variations in their collagen domain or CPP domain. Table 2 lists the properties of each peptide that was tested. The results show that peptide does not need to be folded into triple helix to interact with the FaDu cell surface (FL6V1), but they need a CPP domain—either RRGRRG (1) or R_6_ (2)—that carries a positive charge (Figure 1). It was unexpected that FLV2R did not interact with the cell surface while FL8V2 did (Figure 1). The main difference between these two peptides is in their collagen domains: where FL8V2 and FLV2R share the same amino acid compositions in their collagen domain, FLV2R’s domain has a scrambled sequence. Since FL6V1 does interact with cell surface, it seems that the peptide needs a collagen sequence, (POG)_n_, but not necessarily in a specific configuration, and needs either CPP sequence (RRGRRG or R_6_) to adsorb on the FaDu cell surface. This behavior suggests that deposition is related to the presence of interactions that are driven by electrostatic forces, but could be specific to some degree, hence the need for the presence of a (POG)_n_ sequence in the collagen domain.

We considered two proteins that are known to be upregulated and present at the FaDu cell surface that are not overexpressed in other cancer cell lines that we have studied in the past: junctional adhesion molecule A (JAM-A), expressed in tight junctions (TJ), and heparan sulfate proteoglycans (HSPG), which result from the downregulation of heparanase. The colocalization studies between the JAM-A and FL8V1 show lack of spatial overlap between the two (Figure 2), which is confirmed with the low Pearson’s correlation coefficient of 0.142. Thus, the increased concentration of hybrid peptides on the cell surface is not related to JAM-A surface localization. In the event that the hybrid peptides interact with other TJ proteins (i.e., zonula occludens or claudins), colocalization experiments involving JAM-A protein and the hybrid peptide should give positive results as all TJ proteins are localized in close proximity [30]. Thus, we can conclude that the hybrid peptides visible on the cell surface are not present in TJ.

The HSPG are known docking sites for protein and peptides and promote cell surface adhesion. The presence of heparan sulfate (HS) introduces a negative charge to cell surface due to presence of sulfate groups; thus, the large positive charge present on CPP domain of hybrid peptides could be susceptible to binding with sulfate groups. In addition, the need for the collagen sequence in hybrid peptides (Table 2) to adsorb on the FaDu cell surface also points out a potential interaction with HSPG. Indeed, the salt wash of the cells was able to remove the hybrid peptides from the cell surface, and the heparin column experiment confirmed that the peptide is able to bind to HS. We have not observed this behavior in other cell lines, but out of all the previously studied cell lines, only FaDu were identified in the past as carriers of T5 heparanase splice variants that produce protein lacking the enzymatic activity of heparanase. This would cause an increased concentration of HS on the FaDu cell surface. We believe that the interactions between the HS and COL/CPP peptide carrier are not very strong because 2%KCl (0.268 M) is able to effectively remove the peptide from the cell surface (Figure 3). Moreover, the incubation of FL8V1-treated FaDu cells with suramin, which contains six sulfonic acid groups, was able to significantly reduce the amount of peptide present at the cell surface (Figure 4). The adsorption of FL8V1 on the cell surface is a reason for concern, if hybrid peptides are to be used as drug carriers for cancer drugs. Nevertheless, following the salt wash or suramin treatment, confocal microscopy images (Figure 3 and Figure 4) clearly indicate the presence of endosome-like structures, so even if the entry of some peptide carriers is halted at the cell surface, the remaining carriers are able to cross the membrane.

The colocalization data (Figure 5) between EEA1 and FL8V1 shows that the COL/CPP peptide are entering the cell via endosomal pathway (Pearson’s correlation coefficient = 0.691) and are able to escape from the endosome before it transforms into lysosome. This conclusion is supported by the lack of colocalization between LAMP1 and FL8V1 (Pearson’s correlation coefficient = 0.296). Furthermore, when the COL/CPP peptide carrier is modified with paclitaxel (PTX8V1), the IC_50_ (Figure 6) measured in FaDu cells is 580 nM, which is about 20 times higher than values expected for PTX [7]. While this is not the desired efficacy of PTX, the effectiveness of PTX8V1 is substantial. In conclusion, the deposition of COL/CPP hybrid peptides on the FaDu cell surface is most likely related to presence of HSPG and while it lowers the efficacy of PTX, it does not preclude the application of COL/CPP hybrid peptides as carriers for cancer drugs to FaDu. This limitation of the delivery of paclitaxel with hybrid peptide carriers to FaDu cells may be significant in the future design of HNC treatments.

## 4. Materials and Methods

### 4.1. Peptides

Peptides were purchased from the Tufts University Core Facility (Medford, MA, USA), solid support synthesis and HPLC purification) unmodified or modified with fluorescein isothiocyanate (FITC; excitation wavelength (EX) = 494 nm, emission wavelength (EM) = 521 nm) at the *N*-terminus via a β-alanine–glycine–glycine linker. Both terminals were blocked; the *N*-terminus by acylation and C-terminus by amidation.

Peptide PTX8V1 was synthesized in two steps by bioconjugation with paclitaxel via primary amine of lysine in position 14. The details are described elsewhere [11]. In short, following Deutsch’s method, paclitaxel and succinic anhydride were combined to form 2’-succinyl-paclitaxel. Subsequently 2’-succinyl-paclitaxel was conjugated to peptide via HATU coupling. The synthesis was confirmed with NMR, electrospray ionization mass spectrometry (ESI-MS), and HPLC, available to view in supplemental material in Ayalew et.al. [11].

All stock peptide solutions (PBS, Corning, pH 7.3–7.5) were annealed by preheating to 85 ± 5 °C for at least 20 min and allowed to cool to room temperature. Subsequently, peptides were diluted to varying final concentrations (10–50 µM) in Eagle’s Minimum Essential Medium (EMEM 1× Corning).

### 4.2. Cell Culture

The hypopharyngeal squamous cell carcinoma cell line FaDu was obtained from the American Type Culture Collection (ATCC, Manassas, VA, USA). FaDu was cultured according to the ATCC specifications; EMEM was supplemented with 10% Fetal Bovine Serum (FBS, HyClone, South Logan, UT, USA), with the addition of 0.5% Penicillin Streptomycin l-Glutamine mixture (PenStrep, Lonza, Basel, Switzerland) to prevent mold and bacterial growth. The FaDu cells were cultured in T-25 flasks until at least 80% cell confluency was achieved.

### 4.3. Confocal Microscopy

Cells were imaged using a confocal microscope (Olympus FluoView 1000, Tokyo, Japan). FaDu Cells were seeded at a density of 50,000 cells/mL in complete media in 35-mm Petri dishes with No. 1.5 coverslip as a bottom (MatTek Corporation, Ashland, MA, USA) and incubated overnight. Cells were incubated with peptides in media (10–50 μM) for 30–60 min. After incubation, cells were washed two times. If nuclear dye was used, a Hoechst (Thermo Fisher Scientific, Waltham, MA, USA) nuclear stain solution diluted to 1:2000 was added to the cell culture and incubated for 10 min at 37 °C. The Hoechst stain was removed, and the cell culture was washed at least once with PBS. EMEM medium was added and the cell culture was imaged immediately.

### 4.4. Colocalization of JAM-A and FL8V1

FaDu cells were seeded onto a MatTek dish (No. 1.5, 35 mm). After the cell culture was at least 70% confluent, the old media was removed. The cell culture was washed gently with PBS and a FL8V1 solution (10 µM) was added. The cells were incubated at 37 °C for 30 min. Subsequently, the FL8V1 solution was removed and the cell culture was washed with PBS. Colocalization was performed using rabbit JAM-A antibody (Thermo Fisher Scientific). Rabbit anti-JAM-A antibody (1:4) was diluted to a final dilution of 1:100 in PBS. Alexa Fluor 594 conjugated goat anti-rabbit IgG (Cell Signaling Technology) was used as a secondary antibody (1:500). The cell culture was imaged immediately using the Olympus FluoView confocal microscope (λEX = 488 and 559 nm for FITC tag and Alexa Fluor 594 excitation, respectively) using the 60× oil immersion objective. Colocalization analysis was performed using Image J (NIH, open source software, Bethesda, MD, USA).

### 4.5. Colocalization of EEA1 or LAMP1 and FL8V1

FaDu cells were seeded onto a MatTek dish (No. 1.5, 35 mm). After the cell culture was at least 70% confluent, the old media was removed. The cell culture was washed gently with PBS and a FL8V1 solution (10 µM) was added. The cells were incubated at 37 °C for 30 min. Subsequently, cells were washed with 2% or 4% KCl solution (10 min at 37 °C) and PBS. Colocalization was performed using Rabbit Early Endosome Antigen 1 antibodies (EEA1) antibody (Cell Signaling Technology, Inc., Danvers, MA, USA) or anti-late endosomal/lysosomal marker: Lysosome Associated Membrane Protein 1 (LAMP1) (D2D11) XP rabbit monoclonal antibody (1:500). The Alexa Fluor 594 conjugated anti-rabbit IgG (1:200) (Cell Signaling Technology, Inc., Danvers, MA, USA) was used as a secondary antibody. The cells were imaged with Olympus FluoView 1000 confocal microscope (λEX = 488 and 559 nm for FITC tag and Alexa Fluor 594 excitation, respectively) using the 60× oil immersion objective) and colocalization analysis was performed using Image J (NIH).

### 4.6. Salt Wash

A solution of 5% KCl was prepared and passed through a 0.22 µm sterile filter. FaDu cells were seeded into MatTek dishes (No. 1.5 coverglass, 35 mm). After the cells were at least 70% confluent, the media was removed and the cells were gently washed with PBS. A FL8V1 peptide solution (10 µM) was added and the MatTek dish was placed in an incubator (37 °C) for 20 min. The old medium was then removed and the cell culture was gently washed with PBS twice. A solution of 5% by weight KCl was diluted to final concentrations of 2% or 4%. FaDu cells were incubated with KCl solution at 37 °C for 10 min. The salt wash was discarded; cells were washed twice with PBS. Additional EMEM medium was added before the cell cultures were imaged using confocal microscopy (λ_EX_ = 488 for FITC tag) using the 60× oil immersion objective.

### 4.7. Suramin Wash

FaDu cells were seeded into MatTek dishes (No. 1.5 coverglass, 35 mm). After the cells were at least 70% confluent, the media was removed and the cells were gently washed with PBS. The cells were incubated with (1) FL8V1 (EMEM, 50µM, 30 min, 37 °C) washed and subsequently incubated with suramin (Santa Cruz Biotechnology, Dallas, TX, USA; EMEM, 100 µM, 30 min, 37 °C); or (2) incubation order was reversed (suramin, then FL8V1). The cells were washed twice with PBS. Additional EMEM medium was added before the cell cultures were imaged using confocal microscopy (λ_EX_ = 488 for FITC tag) using the 60X oil immersion objective.

### 4.8. IC_50_ Assay

To measure IC_50_, a CellTiter 96^®^ Non-Radioactive Cell Proliferation Assay (MTT) from Promega was utilized for all cell lines. For FaDu cells, PBS and trypsin were used to induce detachment. In a 96-well plate, cells were seeded at a density of 2.5 × 10^5^ cells per well. Subsequently, concentrations (1 nM–50 uM) of the stock solution of PTX8V1 were added to the plate and incubated for 48 h. Next, MTT dye was added to each well and incubated at 37 °C for 4 h. This followed with the addition of solubilizing solution and an overnight incubation period at 37 °C. The plate was read through photometric scans at 570 nm using the Thermo Scientific Varioskan Flash Reader (Waltham, MA, USA). From the absorbance readings, the average absorbance of each concentration and cell fraction was calculated. Kaleidagraph was used for statistical analysis of data and IC_50_ was determined from curve fitting (sigmoidal) parameters. The sigmoidal line fit equation is defined as (where c is the constant and m is the slope):

(1) $$y=x_{\max}+\frac{\left(x_{\min-}x_{\max}\right)}{\left(1+10^{\left(\log(IC_{50})-c)*m\right)}\right)}$$

### 4.9. Heparin Binding

Heparin-agarose Type I column (Sigma) was equilibrated with 0.01 M Tris-HCl, pH 7.5 buffer. A solution of FL8V1 peptide (20 mg/mL) in 0.01 M Tris-HCl buffer was loaded into the column and eluted with increasing concentration of KCl (0.1–1.5M) in 0.01 M Tris-HCl buffer.

## 5. Conclusions

In this work, we have shown that the efficacy of paclitaxel delivered with collagen/cell penetrating hybrid (COL/CPP) peptide carriers to the hypopharyngeal carcinoma (FaDu) cells is impacted by the cell surface properties. While the CPP domain was design to facilitate the cellular uptake, in the case of FaDu cells, CPP domain enables the adsorption of the carrier on the cell surface. We have determined that the most likely cause is the presence of heparan sulfate proteoglycans (HSPG) on the cell surface, due to overexpression of nonfunctional heparanase. The excess of negatively charge heparan sulfate promotes electrostatic interactions with positively charged CPP domain of the carrier, and limits the hybrid peptide uptake. As far as we observed, this behavior is specific to FaDu cells. Despite surface adsorption, hybrid peptide carriers are able to internalize and release paclitaxel with IC_50_ = 0.6 µM. Therefore, COL/CPP hybrid peptides should still be considered as viable carrier of paclitaxel to FaDu cells, but the limitations associated with presence of HSPG on cell surface has to be taken into account, in the future design of HNC treatments.

## Figures and Tables

**Table 1:** Peptide sequences ^1^.

Peptide	Sequence
FL6V1	FITC-BaGG-POGPOGPOGPOGPOGPOG-RRGRRG
FL8V1	FITC-BaGG-POGPOGPOGPOGPOGPOGPOGPOG-RRGRRG
FL8V2	FITC-BaGG-POGPOGPOGPOGPOGPOGPOGPOG-RRRRRR
FLV2R	FITC-BaGG-GPPOOGPGGGPOOPGOOPGOOPGGOOPP-RRRRRR
FL6	FITC-BaGG-POGPOGPOGPOGPOGPOG
PTX8V1	POGPOGPOGPOG-PK[PTX]G-POGPOGPOGPOG-RRGRRG

^1^ Ba represents β-alanine, O represents hydroxyproline. Dark gray marks carried probe (fluorescein or paclitaxel). Light gray marks CPP domain. All unmarked residues are part of the collagen domain; FLV2R collagen domain is randomized. Peptide nomenclature: Tags (FL = fluorescein, PTX = paclitaxel); number = number of POG repeats in COL domain; V1/V2 = vector 1 (RRGRRG) or vector 2 (RRRRRR) in CPP domain.

**Table 2:** Properties of collagen/CPP hybrid peptides.

Peptide	Helix @ 37 °C	(POG)_n_	CPP	Cell Surface ^1^
FL6V1	No	Yes	Yes (1)	Yes
FL8V1	Yes	Yes	Yes (1)	Yes
FL8V2	Yes	Yes	Yes (2)	Yes
FLV2R	No	No	Yes (2)	No
FL6	Yes	Yes	No	No

^1^ Refers to observed deposition of peptide on the FaDu cell surface.
